# Editorial: Advanced Materials for the Restoration and Reconstruction of Dental Functions

**DOI:** 10.3389/fbioe.2021.756860

**Published:** 2021-09-03

**Authors:** Junchao Wei, Jianxun Ding, Bing Han, Chider Chen

**Affiliations:** ^1^ School of Stomatology, Nanchang University, Nanchang, China; ^2^ Key Laboratory of Oral Biomedicine, Nanchang, China; ^3^ Key Laboratory of Polymer Ecomaterials, Changchun Institute of Applied Chemistry, Chinese Academy of Sciences, Changchun, China; ^4^ Department of Orthodontics, School and Hospital of Stomatology, Peking University, Beijing, China; ^5^ Department of Oral and Maxillofacial Surgery and Pharmacology, School of Dental Medicine, University of Pennsylvania, Philadelphia, PA, United States

**Keywords:** biomaterial, dental material, antibacterial material, implant, drug delivery, bone regeneration, tissue engineering

Various dental diseases, such as periodontal infection, endodontic diseases, dentition and tooth defects, malocclusion, maxillofacial trauma, and oral squamous cell carcinoma, have caused severe suffering to patients. To solve these problems, many effective treatments, such as periodontal scaling, root canal therapy, orthodontic treatment, and tooth implantation, have been used in clinic. During most of these treatments, it must be noted that dental materials have played a critical role in realizing the restoration or reconstruction of dental functions.

Dental materials have brought many advanced choices for clinicians to treat various oral diseases and orthopedic aesthetics, thus significantly improving the oral health of human beings. Up until now, different types of materials, including polymers, inorganic materials, and metal materials, have been widely used for dental restoration, dental implantation, fracture fixation, orthodontic appliances and retainers, removable denture, and so forth. To fulfill the clinical requirements, materials with unique properties, such as antibacterial, osteoinductive, bioactive, adhesive, and mechanical properties, are well investigated. Furthermore, some innovative materials, which can be used for controlled drug delivery, are also helpful to treat oral and dental diseases. Besides, biocompatible scaffolds have gained more attention in the dental tissue engineering fields.

In the current Research Topic, the preparation and applications of advanced materials for the restoration and reconstruction of dental functions were contributed by 134 authors, containing seven review articles and 11 research articles (total views 36,348; as of August 25, 2021). These studies developed advanced polymers, inorganic materials, and metal materials for antibacterial, drug delivery, tissue regeneration, and so forth.

The formation of biofilm after the bacterial infection has been a significant headache for patients and physicians, and thus it is essential to investigate the antibacterial dental materials. Norspermidine (NSPD), a kind of polyamine, was a potential anti-biofilm agent, and its effect on mature biofilm of *Candida albicans* and human dental pulp stem cells was investigated (He et al.), demonstrating that the anti-biofilm effect of NSPD was dose-dependent. Microporous materials, such as zeolite, metal-organic frameworks, and covalent organic frameworks, have potential antibacterial properties and their application to treat oral infection diseases, including dental caries, periodontitis, peri-implantitis, endodontic infections, and so forth, were reviewed (Wan et al.). Moreover, the microporous materials could be used as carriers to deliver antibacterial agents, which were widely used as dental materials. Except for antibacterial agents, the delivery of other bioactive agents by various materials, such as polymer/inorganic nanoparticles, liposomes, nanolipids, and hydrogels, is also essential for treating oral cancers. Typically, the state of the art of targeting drug delivery systems for oral delivery of different therapeutics was summarized, and the improvement of clinical efficiency, well-control of drug release, and reduction of side effects of drugs are still significant challenges (Zhang M. et al.).

Materials-mediated bone and dental tissue regeneration is also a hot topic. Tissue engineering has been a promising approach to regenerating the defect tissues and reconstructing the functions, while scaffolds are critical in practical tissue engineering. The injectable chitosan-based hydrogels with various stimuli-responsiveness and payloads have been widely investigated as tissue engineering scaffolds in dental repairs, such as periodontal and dental-pulp regeneration (Tang et al.). Bioactive glass has shown great potential in bone regeneration. Herein, a kind of borosilicate bioglass scaffolds was prepared, and their biodegradation and osteogenesis for mandible reconstruction were well investigated (Zhang P. et al.). Besides, platelet-rich fibrin (PRF) has become an attractive candidate in instrument implantation and regenerative medicine and was used to prepare scaffold blended with poly(vinyl alcohol) and sodium alginate through electrospinning (Nie et al.). PRF could enhance the proliferation and osteogenesis of osteogenic precursor cells, and thus the composite scaffolds can be a promising candidate for bone tissue engineering. Although tissue engineering has aroused much attention, great efforts are still required to expand the clinical applications.

Tissue engineering is a promising approach to attenuate symptoms of temporomandibular disorder (TMD), even repair or potentially replace the injured temporomandibular joint (TMJ) discs. Poly(ε-caprolactone)/polyurethane (PCL/PU) scaffolds were prepared *via* three-dimensional (3D) printing technology to imitate the region-specific biomechanical properties of TMJ discs (Yi et al.). Generally, 3D printing technology has been well used to process dental materials. For example, a metal crown for dental restoration could be prepared through 3D printing, while quality control is a challenge for dental metal. Herein, a combination of 3D scanning and 3D measurement for 3D inspection of the metal crown was proposed, which may archive 3D model and achieve rapid and high-quality control (Du et al.).

The surface performances of materials always determine their bioproperties and affect their functions. In this Research Topic, several studies focused on the surface management of dental materials and investigated their osteogenic properties. Titanium is one of the essential metal materials and widely used as a dental implant, and the surface morphology properties along with compositions may significantly affect its application. The osteointegration and osteogenic properties of titanium implants with hierarchical micro-nano topographies were investigated (Zheng et al.; Shu et al.). Furthermore, graphene-coated titanium was also prepared and used to adsorb local growth factors and promote osteogenic differentiation of bone marrow stromal cells (Lu et al.).

Polyetheretherketone (PEEK) was approved by the United States Food and Drug Administration (FDA) as an implantable biomaterial and has excellent potential for fixed-definition dental bridge brackets, implant abutments, and implants. However, PEEK has insufficient biological activity when used as an implant and cannot form good osseointegration with the surrounding bone tissue. For that, various techniques, such as plasma treatment and polydopamine coatings, have been developed to modify PEEK to improve the bondability between PEEK and teeth (Teng et al.). Besides, Zhang and coworkers summarized the current modification methods for PEEK, including surface modification and blending modification (Ma et al.). Furthermore, inspired by the component, structure, and function of bone tissue, biocompatible and multifunctional PEEK implants could be fabricated (Gu et al.).

There are also other kinds of materials, such as hemostatic and adhesion materials, which are also very essential as dental materials. Both synthetic polymer- and natural polysaccharide-based multifunctional adhesives could be used for hemostasis (Li et al.). Besides, there are also many other requirements for dental adhesives to restore the function of teeth, and the basic adhesion theory and application of adhesion in dental bonding materials were presented (Zhao et al.).

The immune response toward nanomaterials has been an essential factor affecting the reconstruction of functional tissue. Furthermore, the surface morphology, structure, and composition may significantly affect the immune response. Wu and colleagues prepared nanoscale zirconia nanoparticles with various surface performances and investigated their effect on macrophage phenotypes and gingival fibroblasts, and the macrophage modulation could provide a favorable immune microenvironment for soft tissue cell integration (Wu et al.).

The biomechanical properties of dental implants are another essential factor for the restoration and reconstruction of dental functions. Finite element simulation was used to simulate the mechanical behaviors of implants and surrounding bone tissue, which showed that the bone could achieve optimal ingrowth into the gradient porous structure (Liu et al.).

Overall, this Research Topic covered several cutting edge fields in materials used for restoration and reconstruction of dental functions, such as antibacterial materials, bone tissue engineering scaffolds, implants, and so forth, which may help readers get more about the development of dental materials. Furthermore, the works may connect the material scientists and physicians and promote the studies from theoretical investigation to clinical transformation.

